# Physical activity and sleep changes among children with obesity during a period of school closures related to the COVID-19 pandemic

**DOI:** 10.21203/rs.3.rs-3293474/v1

**Published:** 2023-09-13

**Authors:** Jessilyn Dunn, Karnika Singh, Sarah Armstrong, Brooke Wagner, Julie Counts, Asheley Skinner, Melissa Kay, Jennifer Li, Svati Shah, Nancy Zucker, Cody Neshteruk, Lilianna Suarez, William Kraus, Alexandra Zizzi

**Affiliations:** Duke University; Duke University; Duke University; Duke University; Duke University; Duke University; Duke University; Duke University; Duke University; Duke University; Duke University; Duke University; Duke; Duke University

**Keywords:** COVID-19, pediatric health, daily routine disruption, physical activity, sleep, wearable sensors

## Abstract

Daily routines, including in-person school and extracurricular activities, are important for maintaining healthy physical activity and sleep habits in children. The COVID-19 pandemic significantly disrupted daily routines as in-person school and activities closed to prevent spread of SARS-CoV-2. We aimed to examine and assess differences in objectively measured physical activity levels and sleep patterns from wearable sensors in children with obesity before, during, and after a period of school and extracurricular activity closures associated with the COVID-19 pandemic. We compared average step count and sleep patterns (using the Mann Whitney U Test) before and during the pandemic-associated school closures by using data from activity tracker wristbands (Garmin VivoFit *3*). Data was collected from 94 children (aged 5–17) with obesity, who were enrolled in a randomized controlled trial testing a community-based lifestyle intervention for a duration of 12-months. During the period that in-person school and extracurricular activities were closed due to the COVID-19 pandemic, children with obesity experienced objectively-measured decreases in physical activity, and sleep duration. From March 15, 2020 to March 31, 2021, corresponding with local school closures, average daily step count decreased by 1,655 steps. Sleep onset and wake time were delayed by about an hour and 45 minutes, respectively, while sleep duration decreased by over 12 minutes as compared with the pre-closure period. Step counts increased with the resumption of in-person activities. These findings provide objective evidence for parents, clinicians, and public health professionals on the importance of in-person daily activities and routines on health behaviors, particularly for children with pre-existing obesity. We demonstrate the utility of wearable sensors in objectively measuring longitudinal physical activity and sleep behavior patterns in children with obesity and in quantifying changes in their health behaviors due to disruption of structured, daily routines following in-person school closures during the COVID-19 pandemic.

**Trial Registration:**

Clinical trial registration: NCT03339440.

## BACKGROUND AND SIGNIFICANCE

Childhood obesity is a widespread health problem in the United States and is driven by a multitude of factors[1]. Physical activity (PA) and sleep are two especially important modifiable behaviors that support progression toward a healthy weight in children with pre-existing obesity[1, 2]. Structured routines, such as school and extracurricular programs for children, may support consistent engagement in health behaviors. The structured days hypothesis posits that compared to unstructured days, structured school day activities provide opportunities for mandatory PA, reducing screen time, and enforcing waketime.[3] Consistent with this hypothesis, several studies have also reported increased body mass index (BMI) over the summer months as compared with during the school year,[4–7] which further stresses the importance of structure and routine for child health behaviors and ultimately health and weight related outcomes.

The COVID-19 pandemic resulted in unexpected closures of schools and extracurricular programs. Recent literature indicates that for many children, these changes resulted in decreased levels of PA, increased sedentary time, and disrupted sleep patterns.[8–14] During this time, the rate of increase of BMI in children doubled compared to the pre-pandemic period, with larger increases among children with obesity. [15] Children with obesity are already at a higher risk for engaging in less PA and shorter sleep durations[16–19], identifying a critical need for exploring the impact of the COVID-19 pandemic among this population. However, little research addressed PA and sleep routines as a result of the COVID-19 pandemic specifically among children with obesity.

Additionally, a majority of the PA and sleep data in children reported during the COVID-19 pandemic was collected using self-report measures, which have low levels of reliability when assessing PA and sleep patterns,[20, 21] especially for children.[22] Objectively measured PA and sleep from wrist-worn wearable devices can provide more accurate and detailed information, including step counts during specific times of the day and sleep timings. Such objective data have yet to be leveraged to explore the changes in health behaviors that may result from disrupted daily routines (e.g., school and community center closures) due to COVID-19. The findings may inform future decision-making about systematic changes that may affect the daily habits and routines of children with obesity.

Therefore, the purpose of this study was to examine objectively measured PA and sleep in children with obesity prior to, during, and after COVID-19-related closures of in-person school and extracurricular programs.

## MATERIALS AND METHODS

This study is a secondary data analysis of the Hearts & Parks (H&P) crossover randomized controlled trial, a clinic-community collaboration targeted towards reducing childhood obesity among children in North Carolina (NC). Participants were enrolled between February 8, 2018 and March 10, 2020.[23] However, to ensure similar time duration for the statistical comparison between pre-closure and during-closure values, we only include data starting March 1, 2019 for this analysis. Prior to the pandemic, H&P enrolled and randomized 260 children and adolescents with obesity into either the 6-month clinic-community intervention or a waitlist control group, who received usual care until they entered the intervention at 6 months. In the intervention, patients received care at a pediatric weight management program and were able to participate in a structured play and exercise program, Bull City Fit, delivered at a local parks and recreation center. In Bull City Fit (offered 6 days/week), participants engaged in 60 minutes of PA at every session and were offered weekly nutrition education. The waitlist control group was a six-month waitlisted group, where participants received a non-obesity-related literacy intervention during the first six months, after which they were invited to participate in the intervention for the remaining six months. The study was approved by the Duke Health Institutional Review Board (IRB# Pro00086684) and was funded by the American Heart Association (AHA) Strategically Focused Research Network 17SFRN33670990.

The COVID-19 pandemic led to the closure of Bull City Fit in-person sessions as well as in-person school closures beginning on March 15, 2020. For the purposes of our analysis, we define “pre-closure” to be the period between March 1, 2019 and March 14, 2020, “during-closure” to be the period between March 15, 2020 and March 31, 2021, and “post-closure” to be the period between April 1, 2021 and June 30, 2021. These time frames were chosen based on the announcement of stay-at-home orders for North Carolina (announced on Saturday, March 14, 2020)[24], and when most schools in the Durham school district returned to in person learning (April 2021). The inclusion criteria for H&P required that they live in a geographic radius such that the majority of, if not all, children would be attending a public, private, or charter school in Durham County. Given that the number of participants who wore fitness trackers varied over time, sample sizes vary for the different periods.

### Outcomes.

The primary outcomes of interest were PA (defined as step count), bedtime, waketime, and sleep duration pre-, during-, and post- the COVID-19-associated closures.

### Physical Activity.

PA was measured objectively using step counts from a water-resistant Garmin VivoFit 3 wristband, chosen for its long battery life to last for the study duration without the need for charging. Participants were instructed to wear the watch 24 hours a day for the entire one-year study duration. The Garmin Connect app was downloaded and set up on participants’ or their parents’ smartphones. Parents and/or participants were instructed to sync the smartwatches to the app at least once a week. Garmin data were collected and aggregated by Pattern Health Technologies, Inc., who provide digital health platforms to manage health programs. Fifteen-minute epoch-level (where epoch is the time interval for which step count information was provided) step count information was transformed into daily step counts, which was then used to calculate average daily step counts per month (available for n = 252). Zero values were not reported by Pattern Health or Garmin, and thus it was not possible to differentiate non-wear time from sedentary times from the step count data alone. To address this, we leveraged mean motion intensity (MMI), a metric reported by the Garmin device, as a proxy for watch wear. MMI is a proprietary measure provided for each activity epoch that can take on values between 0 and 7, where 0 corresponds to no activity intensity and 7 corresponds to the greatest measurable activity intensity. We defined non-wear as MMI < 1 and used this definition to remove data corresponding to non-wear times from analysis, which resulted in data availability for 218 participants. From there, we only included in this analysis participants who had sufficient step count data covering the entire pre-, during-, and post-closure period between March 1, 2019 and June 30, 2021. Hence, for the subsequent analysis, we employed a data-driven approach and only included data for participants who had more than 60 days of valid data, where a valid day required more than 41% wear time (the presence of more than 40 out of the 96 possible epochs that can be reported in 24 hours) and only considered valid days for analysis. This resulted in data from 94 participants being included in the final PA analysis presented here (female: 55.3%, median age: 9.7 years) (Supplementary Figs. 1 and 2; Table 1), with n = 93 (female: 54.8%, median age: 9.7 years), n = 53 (female: 52.8%, median age: 9.8 years) and n = 8 (female: 50%, median age: 8.8 years) for pre-closure, during-closure, and post-closure, respectively. The post-closure sample size was fairly small in our analysis but we report average PA and sleep metrics post-closure to offer a potential trend in PA and sleep patterns directions.

To account for activities that occurred during school hours, we also explored step counts only during the times when children were expected to be in school (7:00 AM – 4:00 PM on non-summer weekdays). Step count values were extracted on weekdays for all months excluding the summer months of June, July and August, when schools are typically on break.

### Sleep.

Sleep was measured objectively using the Garmin wristband. Sleep epochs were detected by Garmin’s proprietary sleep detection algorithm, and reported in seconds. Sleep epochs varied in duration as expected based on the number of sleep episodes detected and their individual durations.[25] Some nights had multiple sleep epochs for single participants, indicating disturbed sleep, movement during sleep, improper watch wear during sleep, or algorithmic error. The sleep periods labeled by Garmin were used for the sleep duration, bedtime, and waketime analysis.

## DATA/STATISTICAL ANALYSIS

Step data were aggregated at the daily level for each participant by summing the step count within each epoch for each day only for epochs with an associated MMI ≥ 1. The average daily steps per month were calculated for each participant, and then averaged across all participants to calculate the average daily step count per month for the entire cohort. These average values and their standard deviations were reported for each of the three time periods: pre-closure, during-closure and post-closure (n = 93, 53, and 8, respectively), to enable comparison of PA during each period and to explore whether a relationship would emerge between PA and closures. For analysis of PA during school times, step count observations beginning from 7:00 AM and before 4:00 PM on weekdays, for all months except June, July and August, were included. Daily step count (averaged by month) for school times were calculated in the same way as the overall step count analysis described above (n = 93, 52 and 8, respectively for pre-closure, during-closure and post-closure).

For the sleep analysis, we used the time stamp of the earliest sleep epoch recorded after 6:00 PM on that day and 8:00 AM the following day to define the sleep start time, or bedtime. Similarly, we defined waketime as the latest sleep end time among all such epochs. If the bedtime was after midnight, the bedtime date was adjusted to be the date for the previous day. Total daily sleep duration per participant was calculated as the sum of the durations of all sleep epochs for each day. Average bedtime, waketime, and sleep duration per participant were calculated for each month, and these values were averaged for all participants to obtain the overall population average. Average bedtime, and waketime were each rounded to the nearest 15 minutes and were reported, along with the standard deviations, for each of the three time periods: pre-closure, during-closure and post-closure. Additionally, we report summer bedtime and waketime values separately to emphasize similarities in trends during typical summer pre-closure and both school-year and summer months during-closure.

We performed the Mann Whitney U Test to determine whether step count and sleep duration were significantly changed between the pre- and during-closure periods. To account for variations that are potentially attributable to seasonal changes, pre-closure step counts and sleep durations were compared with the corresponding months during-closure. Given that the post-closure data was only available for a small number of participants (N = 8) and for a limited number of months, we exclude the post-closure data from the statistical analysis and only report the average values for all PA and sleep metrics post-closure.

All analyses were conducted using Python version 3.7.4 through Jupyter notebooks (Jupyter notebook 6.0.1) in the Duke Protected Analytics Computing Environment (PACE) given the sensitive nature of the data. The visualizations were generated using the Seaborn library in Python version 3.7.4.

## RESULTS

**Figure 1:**
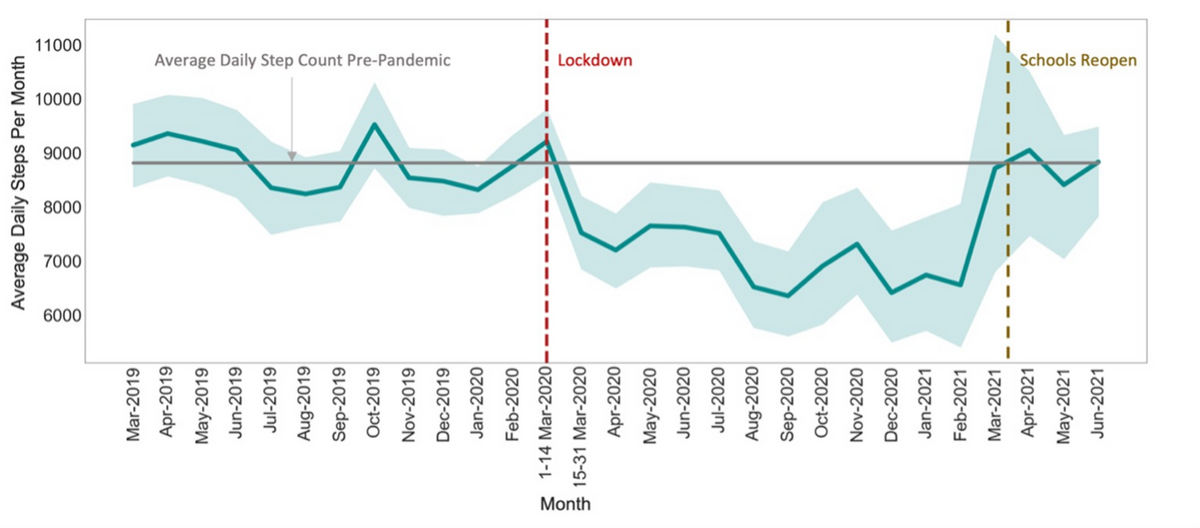
Average daily step count value with 95% confidence interval per month between March 2019 and June 2021. The horizontal grey line indicates the average daily step-count value between March 1, 2019 and March 14, 2020. The dashed vertical red and brown lines indicate the beginning of in-person public school closures and the return to in-person public schools, respectively, in North Carolina.

Of the 94 participants included in this analysis, 52 (55%) were female, 65 (69%) were in the age group 5–10 years (median age 9.7 ± 3.1), and 50 (53%) were assigned to the intervention group. The detailed demographics for study participants who were included in the analysis are shown in Table 1. Pre-closure daily step counts (averaged over the course of the month) ranged from 8,239 to 9,521 steps, with an average of 8,810 ± 453 (n = 93) (Fig. 1). During-closure, the daily step counts dropped significantly from pre-closure to 7,155 ± 669 steps per day (p < 0.05), with an average decline of 1,655 steps (n = 53) (Fig. 1), and ranged from 6,354 to 8,711 steps. Post-closure, the daily step count averaged 8,763 ± 325 steps (n = 8).

**Figure 2:**
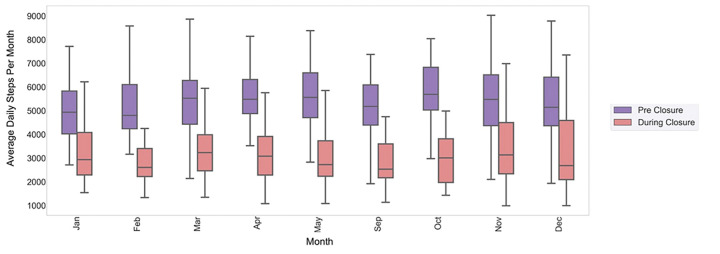
Average daily step count per month between 7 am and 4 pm. A decrease of 1,970 average daily steps observed during the times children were expected to be in school. The purple and pink boxes indicate the pre- and during-closure months respectively.

Pre-closure school-time daily step count ranged from 5,085 to 5,789 steps (average 5,441 ± 229) with an overall average daily step count of 8,889 for in-school months. This step count indicates that roughly 61% of daily steps can be attributed to activity likely occurring during school hours. During-closure, we found that daily step count during school hours declined by 1,973 steps (average 3,468 ± 542, n = 52) (Fig. 2). The post-closure school-time step count averaged 4,616 ± 39 steps (n = 8).

**Figure 3:**
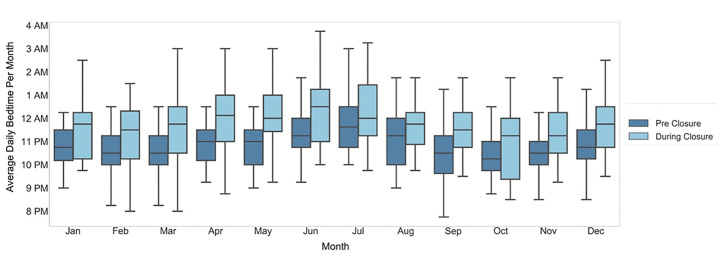
Average daily bedtime pre-closure and during-closure. The dark blue and light blue boxes indicate the bedtime for the pre-closure and during-closure months, which are shown side by side for comparison.

**Figure 4:**
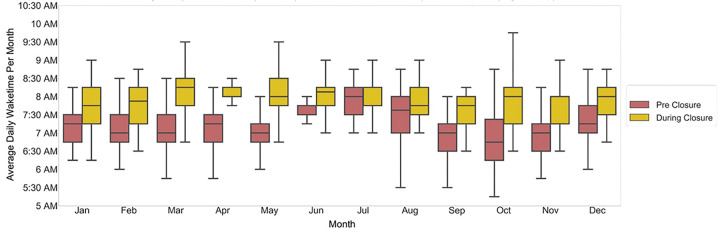
Average daily wake time pre-closure and during-closure. The red and gold boxes show the monthly values for pre-closure and during-closure months, respectively.

**Figure 5:**
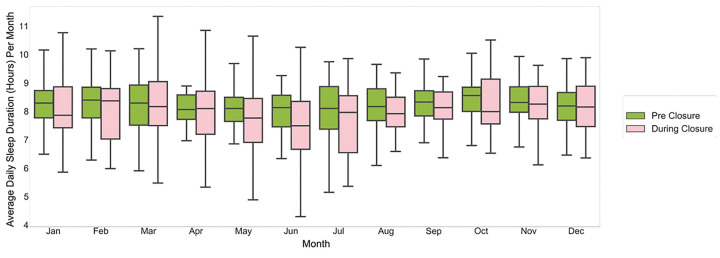
Average daily sleep duration pre-closure and during-closure. The green and pink boxes show the sleep durations for pre-closure and during-closure months, respectively.

We observed a delay in sleep-onset time during-closure (average 11:45 pm ± 32 mins overall, 12:15 am ± 17 mins in summer months) as compared with pre-closure (average 10:45 pm ± 25 mins overall, 11:30 pm ± 17 mins in summer months) (Fig. 3). Similarly, we observed a delay in wake time during-closure (average 8:00 AM ± 11min overall; 8:00 AM ± 3 mins in summer months) as compared with wake time pre-closure (average 7:15 AM ± 16 min overall; 7:30 AM ± 10 mins in summer months) (Fig. 4). Overall sleep duration during-closure decreased by 12 minutes (0.2 hours) as compared with pre-closure, from 8.1 ± 0.17 hours on average to 7.9 ± 0.22 (p = 0.01) (Fig. 5).

## DISCUSSION

This study examined the PA and sleep behaviors of a cohort of children and adolescents with obesity during the COVID-19 pandemic. Children enrolled in this program, aimed at reducing childhood obesity, experienced a sudden and significant decrease in objectively-measured PA and sleep, that correlated in time with COVID-19 related school closures, and resolved when schools reopened. Specifically, we found a group-level decrease in average daily step count of over 1655 steps (19% decrease), delays in sleep onset and waketime by about one hour and 45 minutes, respectively, and decrease in overall sleep duration by 12 minutes (0.2 hours) on an average (a decrease of 2.5%), during-closure compared to pre-closure.

PA and sleep disruption during the COVID-19 pandemic in adult populations has been reported in multiple studies.[26–28] In Fitbit’s analysis of PA data from over 30 million users worldwide there was a decrease of 7–38% in average step count during the week ending March 22, 2020, when compared with 2019.[29] Others demonstrated delayed midsleep times and decreased midsleep variability with stricter lockdown measures in adults across multiple countries using Oura ring sleep tracker data.[30] Similar changes in PA and sleep patterns were expected in children and adolescents as public health guidelines were implemented and structured school activities were suspended in the US.

Various studies in the pediatric population around the world use subjective data to point towards disruption of PA habits and sleep routines, such as reduced PA, increased sedentary times and screen exposure, during the pandemic.[8, 9, 31] Time spent in sports activities decreased by 2.30 ± 4.60 hours/week and sleep time increased by 0.65 ± 1.29 hours/day in 41 children and adolescents with obesity after three weeks into the closures in Verona, Italy.[32] These observations were, however, based on self-reported physical activity, obtained through in-person and telephone interviews. There was a decrease of 435 minutes/week in median time spent in PA in 2,426 children in Shanghai, China, as measured using the Global Physical Activity Questionnaire.[33] Weekly and daily MVPA minutes declined by 30.59 and 15.32 minutes, respectively, based on self-reported assessments and Actigraph data from one week each of pre and post pandemic onset, in children from Italian primary school.[34] However, there is a paucity of longitudinal, objective data about the effect of the pandemic on daily habits in children overall, and especially in children with obesity, who need regular PA and consistent sleep routines. A study measuring step counts among children with congenital heart disease (CHD) using Fitbit Charge 2 devices found a marked decrease in step counts in late March and early April 2020, compared with 2019 and early March 2020.[35] However, this analysis was restricted to children with CHD and was not reported as the lockdown measures continued and eventually relaxed throughout 2020 and early 2021.

Compared to subjective data, information collected from fitness trackers over time is a more reliable indicator of changes in PA and sleep during the pandemic. A major advantage of this study is the availability of continuous, longitudinal data collected from 94 children starting from March 2019 and continuing into 2021, which enabled an objective demonstration of longitudinal changes in PA and sleep in children and adolescents with obesity as a result of the disruption of daily routines due to COVID-19.

Our findings of reduced activity during school times during- pandemic likely point to a relationship between remote learning and sedentary time, and intuitively suggest a reduction in physical activities that would occur during in-person school such as walking to and from school, physical education or recess sessions, and walking between classrooms. While these data represent an association and cannot prove causation, school-based activities have been shown to help children achieve daily moderate-to-vigorous PA (MVPA) goals and maintain timely sleep routines.[36] Structured weekday activities during school days induce healthy PA, sleep regularity, and eating behaviors in children as compared to vacation days or weekends and this might contribute to increased weight gain in children over the summer months as compared with the school year, as documented by some studies.[3, 7] The timing of PA and sleep habit changes reported here correlates very closely with the local school closures and re-openings. Given these factors, we believe that these important health behavior changes may have been an unintended consequence of the disruption of daily routines caused by measures that closed schools and recreational activities during the COVID-19 pandemic.

The behavioral changes we observed are critically important to child health and may have been especially damaging to children with pre-pandemic obesity and cardiometabolic co-morbidities. For example, PA is both a prevention and a treatment strategy for excess adiposity in children. The World Health Organization (WHO) conducted a systematic review in 2020, and found strong evidence that at least 60 min of MVPA per day is associated with lesser adiposity, improved cardiometabolic health, and important cognitive-related outcomes including mood, academic performance, and quality of life.[37] The American Academy of Pediatrics recommends that children with obesity increase their energy expenditure, which may reduce or maintain BMI, and also may improve cardiometabolic health even in the absence of weight loss.[38]

Children’s sleep habits are also known to be related to overall health.[39, 40] An international systematic review including over 500,000 participants from 40 countries showed that longer sleep duration was associated with lower adiposity, better emotional regulation, better academic achievement, and better quality of life.[39] Further, there is a positive association between short sleep duration and greater BMI in children[2]; and healthy sleep, activity, and eating routines are all key components of obesity treatment in children.[41]

This study has strengths and limitations. This study represents a secondary data analysis of the H&P clinical trial, which was collecting objectively-measured PA and sleep data when the COVID-19 pandemic occurred. Data collection was remote, using the wearable Garmin devices; thus measures were able to be continually tracked over the period of the COVID-19 pandemic. Uniquely, this allowed investigators to have the ability to correlate changes in daily PA and sleep with local policies that established mitigation measures, including school closures.

Compared to subjective data, data collected from fitness trackers over time is a more reliable indicator of changes in PA and sleep during the pandemic. A major advantage of this study is the availability of continuous, longitudinal data collected from 94 children starting from 2019 and continuing into 2021, demonstrating longitudinal changes in PA and sleep in children and adolescents with obesity during the COVID-19 associated lockdown measures in the US, as objectively measured from commercial fitness trackers.

The study population was comprised of children and adolescents with obesity enrolled in an intervention study for encouraging healthier lifestyle and PA in Durham, NC. A limitation of this analysis is the availability of data from 94 children and adolescents with obesity from the Durham, NC region only, some of whom were part of the intervention arm of the H&P program, which could have impacted their PA. The number of participants whose data was available for post-closure analysis was also much smaller compared to the overall cohort size. This was because the participants were enrolled for one year in the study and the enrollment ended right before the start of the pandemic. Finally, while fitness trackers provide objective measures of activity as compared with more subjective self-reports, there have been concerns around the accuracy of commercial fitness trackers.[42] One particular challenge for this study is that the tracker did not differentiate between non-wear and sedentary times. However, contextual data was employed to address this concern as described in the methods.

## CONCLUSION

This study suggests there was a decline in PA and disturbed sleep habits among children with obesity during the COVID-19 pandemic. This trend continued for almost a year, with seeming improvements in activity habits back to pre-closure normal with the reopening of schools, pointing to a possible association between structured school activities and healthy PA levels. Findings support the importance of structured, daily routines on promoting health behaviors and, as such, may inform future policy decisions about school and extracurricular activity closures. With this study, we provide information to the community, including teachers, clinicians, and policy makers on how interruptions to normal daily routines including in-person school and activities may impact children. Our results should provide motivation for increasing opportunities for children, especially for those with obesity, for structured activities to promote adherence to routines and prevent unhealthy PA and sleep habits during school closures, such as summer months.

## Figures and Tables

**Table 1:** Participant Demographics

	Overall n (%)	Pre-closure n(%)	During-closure n(%)	Post-closure n(%)
**Age Groups (years)**				
5–10	65 (69%)	64 (69%)	37 (70%)	5 (63%)
11–13	16 (17%)	16 (17%)	9 (17%)	0 (0%)
14–18	13 (14%)	13 (14%)	7 (13%)	3 (37%)
**Gender**				
Male	42 (45%)	42 (45%)	25 (47%)	4 (50%)
Female	52 (55%)	51 (55%)	28 (53%)	4 (50%)
**Race**				
Other	37 (39%)	37 (40%)	21 (40%)	0 (0%)
Black/African American	32 (34%)	31 (33%)	21 (40%)	4 (50%)
White	21 (22%)	21 (23%)	9 (17%)	2 (25%)
Multiracial	2 (2%)	2 (2%)	0 (0%)	0 (0%)
Native Hawaiian/Pacific Islander	1 (1%)	1 (1%)	1 (2%)	2 (25%)
Missing	1 (1%)	1 (1%)	1 (2%)	0 (0%)
**Ethnicity**				
Not Hispanic	51 (54%)	50 (54%)	27 (51 %)	5 (63%)
Hispanic	43 (46%)	43 (46%)	26 (49%)	3 (37%)
**Treatment Group**				
Intervention	50 (53%)	49 (53%)	26 (49%)	5 (63%)
Waitlist Control	44 (47%)	44 (47%)	27 (51 %)	3 (37%)
